# Supplementary material for: Notch1 mutations drive clonal expansion in normal esophageal epithelium but impair tumor growth
Source: Nat Genet. 2023 Jan 19;55(2):232–45. doi: 10.1038/s41588-022-01280-z (PMC9925379; doi:10.1038/s41588-022-01280-z)
Supplement: Supplementary file 1 — Supplementary Note. [file 41588_2022_1280_MOESM1_ESM.pdf]

nature genetics

Article https://doi.org/10.1038/s41588-022-01280-z

Notch1 mutations drive clonal expansion in normal esophageal epithelium but impair tumor growth

In the format provided by the authors and unedited

Supplementary Information

Contents

Supplementary Note 2

1. Stochastic simulations of clonal dynamics 2

1.1 Previous models of mutant clonal dynamics in esophageal epithelium
1.2 2D Wright-Fisher simulations
1.3 Approximate Bayesian Computation
1.4 Parameter fitting results
1.5 Simulations of haploinsufficiency

2. Identifying *Notch1* mutant clones in mouse lineage tracing and in aging experiments 5

2.1 Resolving *Notch1* mutant clones in clonally induced esophageal epithelium
2.2 Identification of spontaneous mutant clones in aging *Notch1*$^{+/-}$ tissue

3. RNA sequencing 6

3.1 Processing epithelium for bulk RNA sequencing
3.2 Processing of frozen tumors and adjacent tissue for RNA sequencing
3.3 Read alignment and DESeq2 analysis
3.4 Functional annotation and gene set enrichment analysis

4. DNA sequencing: sample processing, depth of coverage metrics and mutation calling 8

4.1 Human sample processing for DNA sequencing
4.2 Mouse sample preparation for DNA sequencing
4.3 Custom bait capture kits and coverage metrics
4.4 Alignment, variant calling, and gene selection analysis

5. Copy number variation and copy neutral loss of heterozygosity 11

5.1 Detection of changes in total copy number
5.2 Detection of allelic imbalance

6. Single cell RNA sequencing 13

6.1 Sample processing
6.2 Alignment and transcript quantification
6.3 Cell type detection
6.4 Analysis of keratinocytes
6.5 Cell condition density analysis using Milo

Supplementary note references 15

Supplementary Note

1 Stochastic simulations of clonal dynamics

1.1. Previous models of mutant clonal dynamics in esophageal epithelium

Murine esophageal epithelium (EE) consists of layers of keratinocyte cells and is maintained by the proliferation of cells in the basal layer (**Extended data Fig. 2a**). Each basal cell division can form a pair of cells that remain in the basal layer and go on to divide, a pair of cells that will differentiate and stratify into the suprabasal layers without dividing, or one cell of each fate. The outcome of each division is random, but in homeostasis the balance between dividing and differentiating cells is maintained (**Extended data Fig. 2a**) [1] so that the overall number of proliferative cells remains constant. Due the random (stochastic) nature of the process, some cell lineages will be lost from the tissue and others will form large clones of the original cell [1,2].

A genetic mutation in a basal cell may introduce a bias towards the production of more proliferating cells, leading to the non-neutral expansion of a mutant clone [3,4]. However, in normal EE, a clone cannot not expand indefinitely [5,6]. Mutant clones compete for the limited space in the tissue, and expanding clones revert towards more neutral growth once they encounter similarly fit clones [6]. Therefore, the fate of a cell division depends not only on the dividing cell, but also on the neighboring cells [6]. This spatial clonal competition in EE has been previously been modelled using stochastic simulations of cellular automata on fixed 2D grids [6].

1.2. 2D Wright-Fisher simulations

In this study, we wished to compare the growth of wild type (+/+) clones, induced heterozygous *Notch1*$^{+/-}$ clones and induced homozygous *Notch1*$^{-/-}$ clones. We were looking for broad differences between growth rates, not for precise characterization of the clonal dynamics of each genotype. We therefore aimed to keep the model simple, with few free parameters, so that the results of parameter fitting for each genotype could easily be compared.

We used simulations based on a Wright-Fisher model [7] constrained to a 2D grid [8]. Each cell, $C_{a,b}$, in the grid represents a cell in the basal layer of the EE. The subscripts denote the cell in location a in the grid and generation b of the simulation. We used a fixed size (500×500 cells) hexagonal grid with periodic boundary conditions.

Each cell has a fitness, $F_{a,b}$. In each step of the simulation, each cell $C_{a,b}$ in the new generation picks a parent cell from its immediate neighborhood $\mathcal{N}_a$ (the six adjacent cells plus the cell in the same grid position) in the previous generation (**Extended data Fig. 4a**). The chance of a cell being picked as the parent depends on its fitness relative to the other potential parent cells in the neighborhood,

$$P(C_{a,b}\ parent\ is\ C_{a',b-1}) = \begin{cases} \dfrac{F_{a',b-1}}{\sum_{j\in\mathcal{N}_a} F_{j,b-1}} & a' \in \mathcal{N}_a \\ 0 & a' \notin \mathcal{N}_a \end{cases}$$

A cell can produce multiple offspring in the next generation and the offspring cells inherit the fitness of their parent cells. In this way, a clone with high fitness can expand over multiple generations (**Extended data Fig. 4a**). At the start of a simulation, a small proportion of cells in the grid were randomly selected to be the induced mutant cells. In each simulation, a single fitness value was assigned to all the mutant cells (see **section 1.4** below). The rest of the cells in the simulation were given a fitness of 1. We tracked the sizes of the clones that grew from the mutant cells.

We based the time between generations for all simulations on the average cell division rate previously measured in the murine EE, 0.27 divisions per day [1]. The fitting results showed that this was appropriate for capturing the average growth of the wild type clones in this model (see **section 1.4** below). However, it should be noted that there is not a strict equivalence between cell division rate and the generation time in the simulations, especially those simulations with non-neutral competition. In the model, each cell division is not explicitly simulated; cells can produce multiple offspring cells per generation and this offspring production depends on the relative fitness of neighboring cells.

For each simulation, we had two parameters that were not fixed: the fitness of mutations and the induced proportion of cells at the start of the simulation. The induction proportion must be considered because as the clones expand they can collide and restrict each other's growth. In a highly induced tissue, fit mutant clones will have less room to expand and will be smaller at later time points than in a sparsely induced tissue. The other parameter, mutant fitness, is the key parameter we wish to compare between the genotypes. It represents how much of a growth advantage the mutant has compared to the surrounding non-mutant cells.

1.3. Approximate Bayesian Computation

To fit the parameters to the data we used Approximate Bayesian Computation (ABC) [9,10]. In this fitting method, prior distributions for the parameters are defined. We used uniform distributions across wide intervals: mutant fitness between 0 and 50, induction between 0% and 10% of cells. A parameter set is randomly drawn from the prior distributions, and a simulation run with those parameters. The simulation results are measured against the observed data using summary statistics. We used the Kolmogorov-Smirnov statistic [11] to compare the simulated and experimentally observed basal clone size distributions. We used 100 simulated clones at each time point, and summed the Kolmogorov-Smirnov statistics calculated for each time point to get the total "distance" between the simulation and the data. If the distance between the simulation and the experimental data is less than a set threshold, those simulation parameters are accepted, otherwise they are rejected. Repeating this process many times with parameter sets randomly drawn from the prior distributions builds up a set of accepted parameters, which approximates the posterior parameter distribution [10].

We used a version of ABC based on sequential Monte Carlo sampling (ABC-SMC) [12]. This reduces the required number of simulations by running multiple generations of simulations, with new generations of parameters based on the successful parameters from the previous generation. The threshold distance for parameter acceptance is reduced with each generation so that the distribution of accepted parameter sets increasingly approaches the target posterior distribution [12]. We used the ABC-SMC implementation from the python package PyABC [13], and ran 15 generations using a population of 1000 particles (the required number of accepted parameter sets per generation).

1.4. Parameter fitting results

Firstly, we see that the yellow fluorescent protein (YFP) labelled clones in the *YFPCreNotch1*$^{+/+}$ mice and the YFP + wild type intensity clones in the clonally induced *YFPCreNotch1*$^{+/-}$ and *YFPCreNotch1*$^{-/-}$ mice have inferred fitness values closely centered on neutrality (fitness=1) (Extended data Fig. 4b, c, Supplementary Table 33). Both *Notch1*$^{+/-}$ and *Notch1*$^{-/-}$ clones are fitter than wild type clones, with *Notch1*$^{-/-}$ clones substantially fitter than *Notch1*$^{+/-}$ clones. Should this genotype–fitness relation also apply in humans, this large increase in fitness conveyed by the second *Notch1* mutation may explain the strong selection and high prevalence of "double hit" *NOTCH1* mutant clones in aged human esophagus [14] (Fig. 1d-f).

The fitting results are consistent with the EdU 48h data shown in Fig. 2k,l. In the model, interaction between highly fit mutant cells and adjacent wild type cells biases the wild type cells towards differentiation and the mutant cells towards division. The higher the fitness imbalance, the stronger the fate bias. The stronger differentiation bias in the wild type cells adjacent to *Notch1*$^{-/-}$ clones (Fig. 2l) is therefore consistent with the higher fitness inferred for the *Notch1*$^{-/-}$ clones.

Although mutant fitness is the key parameter we are interested in, the inferred induction proportion can also provide some useful information. It can suggest where the simple model we are using might be less able to replicate the experimental results, and provide reassurance that the conclusions we are drawing from the inferred fitness values are robust. In the cases of neutral clones (*Notch1*$^{+/+}$ and wild type intensity clones from induced mutant mice) the induction proportion was not constrained by the fitting (Extended data Fig. 4b, Supplementary Table 34), because these clones have the same fitness as the surrounding wild type cells and therefore do not impact the growth of any other clones. The inferred induction proportion for the *Notch1*$^{+/-}$ clones was larger than for the *Notch1*$^{-/-}$ clones (Extended data Fig. 4b, Supplementary Table 34). In simulations of the best fitting *Notch1*$^{+/-}$ parameters, the mutant clones were colliding at the later time points, reducing the clone sizes (Video 1). This high density of clones and extensive clonal collision was not occurring to such an extent in the experimental data (Extended data Fig. 3j, m), suggesting that the model is not fully capturing all details of the *Notch1*$^{+/-}$ clonal dynamics and may be overestimating the fitness of large *Notch1*$^{+/-}$ clones. However, the conclusions of the inferred fitness comparison are still valid: that the *Notch1*$^{+/-}$ clones have a clear growth advantage over wild type cells, and that the *Notch1*$^{-/-}$ clones have much larger growth advantage than the *Notch1*$^{+/-}$ clones.

1.5. Simulations of haploinsufficiency

It is clear from the lineage tracing experiments and the model fitting results that *Notch1* is haploinsufficient, i.e. the heterozygous mutation gives a growth advantage to the clone. This growth advantage of the heterozygous mutant increases the chance of a double Notch1 mutant occurring because a higher proportion of the tissue becomes primed with the first mutation.

To illustrate the large difference haploinsufficiency makes to *Notch1* mutant tissue takeover, we ran simulations of haplo*insufficient* and haplo*sufficient* versions of *Notch1.* In the haplosufficient simulations, the first *Notch1* mutation has the same fitness as wild type cells, and a second *Notch1* mutation in the same cell increases the fitness to the best fitting *Notch-*$^{-/-}$ fitness (see section 1.4). In the haploinsufficient simulations, the first *Notch1* mutation provides a fitness advantage equal to the

best fitting *Notch1*$^{+/-}$ fitness, then a second *Notch1* mutation in the same cell increases the fitness to the best fitting *Notch1*$^{-/-}$ fitness

We ran simulations with a very low mutation rate to parallel the experiments in which we age wild type mice and observed the appearance of *Notch1* mutant clones (Extended data Fig. 4e, f, Video 2). Each cell has a 0.0005% chance of gaining a mutation in each generation, with the mutation rate picked so that the haplosufficient simulations approximate the experimental data (Extended data Fig. 4e). In the simulations, a mutation can either appear on allele 1 or allele 2 of Notch1 with 50% chance of each. A cell with a single mutant allele has the heterozygous fitness of that simulation type (see above), a cell with both alleles mutated has the homozygous fitness. A second mutation to the same allele does not change the cell fitness. We ran the simulations for 5000 days or until all cells in the tissue had both Notch1 alleles mutated.

The simulations showed that it would take substantially longer for the tissue to be swept by the homozygous mutant clones if *Notch1* was haplosufficient (Extended data Fig. 4e, f, Video 2). This shows how the haploinsufficiency of *Notch1* is a key property that allows mutations in this gene to dominate clonal competition in normal EE.

2. Identifying *Notch1* mutant clones in mouse lineage tracing and in aging experiments

2.1 Resolving *Notch1* mutant clones in clonally induced esophageal epithelium

To genetically label wild type and *Notch1* mutant clones in the same tissue, we crossed floxed *Notch1* mice with floxed *Rosa26*^{EYFP} animals carrying a conditional fluorescent YFP reporter at the *Rosa26* locus and the inducible *AhCre*^{ERT} mice (*YFPCreNotch1* triple mutant mice) [1,15]. Excision of the *Notch1* allele and expression of the YFP reporter at the Rosa26 locus can occur in the cells either in combination or separately, resulting in four different population of cells, *Notch1* mutant or not and expressing YFP or not. To discriminate YFP+ *Notch1* mutant clones from YFP+ *Notch1* wild type clones we used NOTCH1 staining. In induced *Notch1*$^{flox/flox}$ tissue, YFP+ clones expressing NOTCH1 were categorized as wild type (+/+), while the clones without detectable expression of NOTCH1 were classified as *Notch1*$^{-/-}$.

To distinguish the wild type and *Notch1*$^{+/-}$ clones in induced *YFPCreNotch1*$^{+/flox}$ tissue, we set up a method based on measurement of NOTCH1 immunofluorescence. YFP expressing clones were imaged with confocal microscopy at 40x objective and analysis was performed using Volocity 6 software (Perkin Elmer). For each clone, NOTCH1 staining was detected in the basal layer (lasso tool selection) and the mean intensity was recorded and normalized to the mean intensity of DNA staining. Close by areas outside the clones were analyzed the same way and used to normalize intensity data between images. The NOTCH1 staining intensity distribution of n=126 YFP+ clones in 3 *YFPCreNotch1*$^{+/+}$ mice was used as a reference. The distribution of the relative intensities approximated to a normal curve (p=0. 25, Shapiro-Wilks test) with mean 1.02 and standard deviation 0.15, fit with Python package Scipy v1.5.2. The distribution of relative NOTCH1 staining intensity of n=172 YFP clones in induced *YFPCreNotch1*$^{+/flox}$ mice (13 weeks p.i.) was significantly different from the reference distribution ($p=5e^{-27}$, two-tailed Kolmogorov-Smirnov test, Supplementary Table 35). To confirm that clones in induced *YFPCreNotch1*$^{+/flox}$ mice were a mixture of *Notch1* wild type and *Notch1*$^{+/-}$ clones, we fit a Gaussian Mixture model with two components to the *Notch1*$^{+/flox}$ relative

intensity distribution using the Python package Scikit-learn v0.23.2. The resultant fit consisted of a normal distribution highly similar the reference distribution (mean=1.0, sd=0.15), and a normal distribution with half the intensity of the reference distribution (mean=0.53, sd=0.15). We chose to split the induced *Notch1*$^{+/-}$ clones using a relative intensity threshold of 0.75. This threshold, slightly lower than the mid-point of the two distributions, was selected so that very few of the wild type clones (<5%) would be categorized as *Notch1*$^{+/-}$. The higher intensity clones were assumed to be *Notch1* wild type (+/+ in +/flox) and had a similar distribution of staining intensity to those in the *Notch1*$^{+/+}$ mice (p=0.38, two-tailed Kolmogorov-Smirnov test). The remaining clones were categorized as *Notch1*$^{+/-}$ (Supplementary Tables 6, 7 and 35). This method was used at each time point of clonal counting and to categorize YFP+ clones and distant or edge cells in clonal EdU experiments. The number of basal and suprabasal cells in each clone was counted. The validity of this method was further confirmed using a qPCR assay measuring the recombination of *Notch1* locus on genomic DNA extracted from microdissected clones (see Method section *'qPCR Recombination assay'* and Extended data Fig. 3).

Clones with no basal cells in the induced mutant mice could not be categorized with this method but represented only ~1% of the clones in the dataset (Supplementary Table 7).

2.2 Identification of spontaneous mutant clones in aging *Notch1*$^{+/-}$ tissue

Esophageal tissue from induced *YFPCreNotch1*$^{+/flox}$ mice aged to 54-78 weeks-old was incubated for 2h30 in 5mM EDTA solution at 37 °C before peeling. Whole mount esophageal epithelium was stained for NOTCH1, YFP and Sytox™ Blue and imaged. The large and ovoid areas negative for NOTCH1 staining suggested the presence of *Notch1* mutant clones (Fig. 3b, e). To identify putative clones expressing NOTCH1, we used the combination of NOTCH1 and YFP staining. In highly induced heterozygous tissue, YFP staining typically revealed a patchwork of small YFP+ clones. Putative clones appeared as large and ovoid areas devoid or fully stained with YFP (Fig.3e). Sequencing performed as in '*Mouse sample preparation for DNA sequencing'* confirmed that this method allowed the identification of large spontaneous clones carrying *Notch1* mutations on the non-recombined allele (Fig. 3e-g). EDTA treatment triggers the cleavage of NICD1, followed by nuclear translocation without ligand activation [16]. We used this assay to test the functionality of the cleavage of the spontaneous NOTCH1 positive clones identified (Extended data Fig. 5e-j).

3. RNA sequencing

3.1 Processing epithelium for bulk RNA sequencing

YFPCreNotch1$^{flox/flox}$ and *YFPCreNotch1*$^{+/flox}$ mice between 10-16 weeks of age were injected intraperitoneally (i.p.) on two consecutive days with β-Naphthoflavone (BNF) at 80 mg.kg-1 and tamoxifen (TAM) at 1 mg and aged for 8 weeks before collection**.** The dose was set to allow *Notch1*$^{+/-}$ or *Notch1*$^{-/-}$ cells to fully occupy the tissue by two months post induction, as measured by qPCR recombination assay, qPCR assay and protein assay (Extended data Fig. 3f-h). Uninduced littermates were used as wild type control. Esophageal tissues were incubated for 15 minutes in Dispase I (Roche catalog no. 04942086001) diluted at 1mg/ml in PBS before separating the epithelium with fine forceps. Peeled epithelium was lysed in RLT Plus buffer using MagNA Lyser Green Beads (Roche, catalog no. 03358941001) and FastPrep 24 homogenizer (MP Biomedicals, catalog no.116004500). Lysates were then processed for RNA and DNA extraction using AllPrep DNA/RNA Mini kit (Qiagen), with Complete Protease Inhibitor (Roche, catalog no. 11836170001) added instead of the proteinase

K step, allowing protein extraction as described in the Immune Capillary Electrophoresis section. Total RNA was measured using Qubit™ RNA BR Assay Kit (Thermo Fisher Scientific, catalog no. Q10211).

For RNA-seq, libraries were prepared from 1 µg total RNA using an Agilent Bravo robot with aKAPA Standard mRNA-Seq Kit (KAPA BIOSYSTEMS). In-house sequencing adaptors were ligated to 100-300 bp fragments of input dsDNA. Samples were then subjected to 10 PCR cycles using sanger_168 tag set of primers. Paired-end sequencing was performed on Illumina HiSeq 2500 with 75 bp read length.

3.2 Processing of frozen tumors and adjacent tissue for RNA sequencing

Flash frozen tumors and adjacent normal tissue collected 28 weeks after DEN and Sorafenib treatment were cut into serial 14 µm cryosections, dried in the cold chamber for ~10 min and fixed/stained/dried as follows: 2min in 70% Ethanol; 2 min in Nuclease free water; 30s in Cresyl Violet solution at 1% (w/v) in 50% Ethanol; 30s in eosin at 0.25% in 50% Ethanol; 30s in 70% Ethanol; 1 min in 100% Ethanol; dipped in fresh 100% Ethanol and air dried for 3 minutes. All processing was performed in nuclease free conditions. To separate the tumor mass and normal tissue from the underlying submucosa, Cresyl Violet/eosin stained sections were immediately micro-dissected using a micro knife (FST, catalog no. 10317-14). Dissected tissue from serial sections was pooled and RNA extracted using the RNAeasy Microkit (Qiagen, catalog no. 74004) following the manufacturer's instructions for purification of total RNA from microdissected cryosections. 6 to 20ng of total RNA was processed using NEBNext rRNA Depletion Kit v2 (NEB #E7400, #E7405) followed by NEBNext® Ultra II Directional RNA Library Prep Kit for Illumina® (NEB #E7760, #E7765) following manufacturer's instructions. Fragmentation was performed for 12 minutes at 94 degrees. The adaptor was diluted 25 fold. 15 cycles of PCR amplification were performed for all libraries. Paired-end sequencing was performed on Illumina HiSeq 4000 with 75 bp read length.

3.3 Read alignment and DESeq2 analysis

Reads were mapped using STAR 2.5.3a, the alignment files were sorted and duplicate-marked using Biobambam2 2.0.54, and the read summarization performed by the htseq-count script from version 0.6.1p1 of the HTSeq framework [17,18] .Differential gene expression was analyzed using the DESeq2 R package [19] and the downstream pathway analysis and visualization using R (https://www.R-project.org/) and the packages Pheatmap (https://cran.r-project.org/package=pheatmap), RColorBrewer (https://cran.r-project.org/package=RColorBrewer), clusterProfiler [20] and org.Mm.eg.db (https://bioconductor.org/packages/org.Mm.eg.db/). Differentially-expressed genes (DEG) are hits reported by DESeq2 with adjusted p value (p_adj) of less than 0.05. DEG between two groups were calculated after running DESeq2 using all samples from all groups in each dataset. For RNA-seq of normal esophageal epithelium (Extended data Fig. 6), background noise was filtered out from the dataset using the following rule: quality control was performed and an outlier control sample was identified; Genes with an eigenvector value of >0.05 or <-0.05 for the two principal components calculated among wild type control samples were removed from the dataset (151 genes out of the initial 47,467 genes) for final analysis. This resulted in removing seven potential false positive hits from the output of DESeq2 analysis (*Acacb*, *Gm14165, Gm8730, Gm9625, Hcar1, Rplp0-ps1* and *Xlr3a*) and did not modify any of the conclusions from the dataset analysis.

Hierarchical clustering was performed using ward.D2 agglomeration method as implemented in the R base package *hclust*. Heatmaps in Extended data Fig. 6d were generated from the ratio of TPM values of the treated sample over the average of the respective control samples.

3.4 Functional annotation and gene set enrichment analysis

The functions of the DEGs from DESeq2 analysis were annotated using the database for annotation, visualization, and integrated discovery (DAVID v6.8) and Gene Ontology Biological processes (GOBP) [21]. Only GOBP categories with p-value<0.05 were considered statistically significant. To draw the bar plots, the –log10 (P-value) of each GOBP was calculated.

When up/down DEG lists were <500 genes, Gene Set Enrichment Analysis (GSEA) was preferred for increased statistical sensitivity[22]. GSEA was performed to identify gene sets differentially expressed in *Notch1*$^{-/-}$ and *Notch1*$^{+/+}$ samples (Extended data Fig. 6e; Fig. 8e, Extended data Fig. 10d, Supplementary Tables 15 and 29) [22]. Read counts were normalized using DESeq2 median of ratios method[23]. Genes with zeros read counts value were ignored. We used GOBP or Kyoto Encyclopedia of Genes and Genomes (KEGG) gene sets v7.5.1 from the Molecular Signature Database (MSigDB) in GSEA software v4.2.3. We used gene set permutations and only gene sets with normalized enrichment score (NES) associated with false discovery rates (FDR) <0.05 were considered statistically significant. GOBP results were plotted in the figures cited above, KEGG results were consistent and are only showed in Supplementary Tables 15 and 29.

4. DNA sequencing: sample processing, depth of coverage metrics and mutation calling

4.1 Human sample processing for DNA sequencing

Sampling

For sequencing 20 µm cryosections were fixed with 4% paraformaldehyde (PFA) for 10 min, stained for NOTCH1 (Cell Signaling catalog no. 3608) using the Rabbit specific HRP/DAB (ABC) Detection IHC Kit (Abcam Plc catalog no. ab64261) and counterstained with Hematoxylin. Intercalated 10 µm thick sections were stained for NOTCH1 (Supplementary Table 31). DNA was extracted from 250-1000 µm of epithelium microdissected from 4 to 6 successive serial sections using a QIAMP DNA microkit (Qiagen) by digesting overnight and following manufacturer's instructions. Esophageal muscle DNA was used as a germline control.

Targeted sequencing

An Agilent SureSelect custom bait set was used (Supplementary Table 1, see below**)**. Human micro-dissected samples were submitted for DNA library preparation. Multiplexed samples were sequenced on an Illumina HiSeq 2000 using paired-end 75-base pair (bp) reads. Total number of protein altering mutations from 86 sequenced samples were from CaVEMan and cgpPindel variant calling algorithms [24,25]. *NOTCH1* mutations shown in Fig.1c-f were generated from these two algorithms with additional calls from the ShearwaterML algorithm [14,26,27], Supplementary Table 4.

4.2 Mouse sample preparation for DNA sequencing

Prior to sequencing, epithelium was processed for whole mount immunostaining and imaged by confocal microscopy. DNA from ears of the same mice was used as germline controls.

Punch sampling

To sequence stained areas of interest, stained and imaged tissue was flattened and sampled under a Fluorescent Stereo Microscope Leica M165 FC (Leica) using 0.25 mm diameter punch (Stoelting, catalog no. 57391) (Fig. 3e, Extended data Fig.5). DNA was extracted using Arcturus® PicoPure® DNA extraction kit (Applied Biosystems, catalog no. 11815-00) following the manufacturer's instructions).

Gridding

Epithelium was flattened and cut into an array of 2 mm^2 contiguous biopsies (Fig. 6a-c, Extended data Fig. 8a,b). Samples were digested and DNA extracted using the QIAMP DNA microkit (Qiagen, catalog no. 56304) following the manufacturer's instructions.

Tumor sampling

Flash frozen tumors were cut into serial 50 µm cryosections and stained for KRT14, KRT6a and Sytox™ Blue (Fig. 6a-c, Extended data Fig. 8a-c), and micro-dissected under a Fluorescent Stereo Microscope Leica M165 FC (Leica) using a micro knife (FST, catalog no. 10317-14). Dissected tumor was pooled and DNA extracted using the QIAMP DNA microkit (Qiagen, catalog no. 56304) following the manufacturer's instructions.

Targeted Sequencing

Samples were multiplexed and sequenced on Illumina HiSeq 2000 sequencer using paired-end 75-base pair (bp) reads. Agilent SureSelect custom bait capture kits were used (Supplementary Table 1).

4.3 Custom bait capture kits and coverage metrics

Custom bait set design

For targeted sequencing experiments, we used three different Agilent SureSelect custom bait capture kits. Lists of the genes included in each custom bait capture kits are detailed in Supplementary Table 1**.** For the Human dataset, we used a kit comprising 322 genes designed to include frequently mutated genes in cancer. For the targeted sequencing of mouse tissue, we used a kit comprising 73 cancer related and Notch pathway related genes. This kit (baitset_3181471_Covered.bed) also includes several regions of chromosome 2, which carries *Notch1* gene. These regions, identified in a preliminary assay, allowed detection of single nucleotide polymorphism (SNP) signal along the chromosome resulting in robust copy neutral loss of heterozygosity (CNLOH) analysis.

Coverage metrics

For gridded tissue and tumor mouse samples, the average depth of coverage across all genes after removing off-target reads, PCR duplicates were: DEN-SOR *Notch1*$^{+/+}$ grids: 478x; DEN-SOR *Notch1*$^{+/+}$ tumors: 768x; DEN-SOR *Notch1*$^{-/-}$ tumors: 741x.

For the samples submitted at low DNA input, the average depth of coverage across all genes after removing off-target reads, PCR duplicates were: Human biopsies: 42x; *Notch1*$^{+/-}$ subclonal biopsies: 194x.

4.4 Alignment, variant calling, and gene selection analysis

Alignment

Paired-end reads were aligned with BWA-MEM (v.0.7.17, https://github.com/lh3/bwa) [28] with optical and PCR duplicates marked using Biobambam2 (v.2.0.86,

https://gitlab.com/german.tischler/biobambam2). Human samples were aligned to the GRCh37d5 reference genome whilst Mouse samples were mapped to the GRCm38 reference.

Mutation calling

Methods used for mutation calling were adapted to the dataset, depending on if the samples were clonal/near-clonal or if we aimed at identifying somatic mutations present in a small fraction of cells within the samples.

Human micro-dissected samples and mouse subclonal samples for clonal or nearly clonal (Fig. 1, Extended data Fig. 1, Fig. 3, Extended data fig. 5c-j, Supplementary Tables 2-4 and 10-11). Substitution mutations were called using the CaVEMan (Cancer Variants through Expectation Maximization, version 1.13.14) variant caller (http://cancerit.github.io/CaVEMan) [24]. Insertions and deletions were called using cgpPindel (http://cancerit.github.io/cgpPindel, version 3.3.0) [25]. Mutations were annotated using VAGrENT (https://github.com/cancerit/VAGrENT, version 3.3.3) [29]. Mutations called in genes that were not listed in the bait kit were removed. Protein altering mutations were only mutations annotated with the effects 'missense', 'nonsense', 'ess-splice', 'frameshift' or 'inframe'. The ShearwaterML pipeline (details below) was also used to analyze the Human dataset in order to report additional calling to the calls identified with CaVEMan and cgpPindel and allow a more exhaustive analysis of *NOTCH1* mutations. Only mutations with variant allele frequency (VAF) ≥0.05 were counted in Fig.1 (Supplementary Tables 2, 4).

Gridded normal tissue and tumors (Fig. 6, Extended data Fig. 8a-c, Supplementary Tables 19, 20) were sampled to analyze gene selection, map somatic mutations clones spanning multiple samples or present in a small fraction of cells within the samples. To do so, we used ShearwaterML algorithm from the deepSNV package (v1.21.3, https://github.com/gerstung-lab/deepSNV) to call for mutation events on ultra-deep targeted data [14,26,27]. Instead of using a single-matched normal sample, the ShearwaterML algorithm uses a collection of deeply-sequenced normal samples as a reference for variant calling that enables the identification of mutations at very low allele frequencies.

The total coverage provided by the references used for each bait kits were as following. For the specifically designed Notch bait kit (Supplementary Table 1), we used a total of 34 germline samples from the ears of the analyzed samples and the ears of extra mice providing a total coverage of 22885x. For the kit used for the Human sequencing, we used 71 germline samples for a total coverage of 14599x.

Gene selection (dNdS)

We used the maximum-likelihood implementation of the dNdScv algorithm (v0.0.1.0, https://github.com/im3sanger/dndscv) to identify genes under positive selection [30]. dNdScv estimates the ratio of non-synonymous to synonymous mutations across genes, controlling for the sequence composition of the gene and the mutational signatures, using trinucleotide context-dependent substitution matrices to avoid common mutation biases affecting dN/dS. Values of dN/dS significantly higher than 1 indicate an excess of non-synonymous mutations in that particular gene and therefore imply positive selection, whereas dN/dS values significantly lower than 1 suggest negative selection.

Mutant tissue coverage

The size of mutant clones within each sample can be calculated by taking into account the area of the biopsy and the fraction of cells carrying a mutation within the sample, as described previously [14,27]. The lower (=VAF) and upper (=2xVAF) bound estimates of the percentage of epithelium covered by clones carrying non-synonymous mutations in a given gene was calculated for each biopsy. The fraction of epithelium covered by the mutant genes was then calculated from the mean of summed VAF (capped at 1.0) of all the biopsies in the same tissue.

Merging of clones

For analysis of the mutational landscape in the normal epithelium from highly mutagenized mice, we performed gridding of the tissue into 2 mm^2 biopsies and kept record of the relative positions of the samples (Fig. 6b,c, Extended data Fig.8a,c). To avoid counting the same mutation multiple times and to obtain a more accurate estimate of clone sizes clonal mutations that spanned between two or more adjacent biopsies were merged as individual events [14,27]. To do this we calculated the mean number of shared mutations between biopsies at increasing distances, since the immediately adjacent samples are predicted to have more shared mutations than distant samples. Mutations common between samples closer than 3 mm were merged.

5. Copy number variation and copy neutral loss of heterozygosity

The *NOTCH1* locus is frequently affected by copy neutral loss of heterozygosity (CNLOH) events [14,27]. CNLOH consists of the presence of two copies of either the maternal or paternal chromosome copy and none of the other. In sequencing data, this manifests itself as a change in the allele frequency of germline heterozygous single nucleotide polymorphisms (SNPs) (typically referred to as b-allele frequency, BAF), whilst the total coverage at the CNLOH event locus remains normal. The detection of a significant change in BAF and the absence of a change in coverage therefore constitutes CNLOH. To detect these events, we applied two pipelines based on previously published methods: One to detect alterations in coverage data and one for germline heterozygous SNPs.

Whilst a change in total coverage can readily be detected in data from both human and mouse samples, typically, inbred mouse strains do not contain enough germline heterozygous SNPs for the purposes of detecting a BAF imbalance. This project, however, contains data sets from mice of mixed-strain background where we did detect the presence germline heterozygous SNPs. We reasoned that, because the mouse strains used in this project are common, any reliable SNP call from the Mouse Genome Project [31], which is an effort to catalogue common strains, could be a viable germline heterozygous SNP candidate. Such a SNP would result from the mixture of strains, or rarely due to a *de novo* germline mutation. We therefore only considered SNPs that were reported by the Mouse Genome Project and subsequently applied stringent coverage criteria to identify heterozygous SNPs in the matched control samples during the analysis (which is detailed further below).

Since both coverage data and germline heterozygous SNPs are available for both species, we applied both pipelines to all human and mouse samples.

5.1 Detection of changes in total copy number

To call changes in total copy number in both human and mouse data we adapted QDNAseq for our purposes [32]. Briefly, QDNAseq typically collects read counts in bins across the genome, normalizes the counts to produce relative coverage (commonly referred to as logR; coverage log ratio), adjusts coverage for GC content correlated artifacts, segments the data in sections of constant signal and finally calls regions that are significantly different from normal as either a gain or a loss.

The standard QDNAseq pipeline was adapted in three ways. First, since the datasets described in this manuscript always include a matched control sample, we included an extra step in the pipeline to adjust for coverage variability observed in the control sample to further reduce noise, as described in [33].

Second, the calling of significantly different regions was adapted with a more conservative test, with the aim to produce robust calls. This step takes as input the segmented genome and each segment is tested by performing two one-sided t-tests: One to test for a gain and one to test for a loss. The test is performed between two distributions, one with the mean and standard deviation calculated from the logR of the segment (H1) and one with a mean of 0 (normal, unaltered logR) and the standard deviation of the segment (H0). This test may detect very small deviations for large segments, in-line with our aim for robust calls, we calculate the mean and standard deviation based on 100 sampled data bins for segments where more than 100 bins are available. The resulting p-values are adjusted for multiple testing (Bonferroni) and 0.05 is used as significance cutoff.

Finally, we included a pre-processing step to allow QDNAseq to run on targeted sequencing datasets. Typically, targeted sequencing data includes read-pairs that fall outside of the targeted genomic areas (referred to as off-target reads) and these off-target reads effectively form a shallow whole genome sequencing sample that can be used to call alterations [34]. To obtain this shallow genome we first count reads in 1kb-sized bins and subsequently remove any bin that overlaps with the targeted genomic coordinates. The remaining 1kb bins are then mapped to 1mb-sized bins, after which the regular pipeline is applied.

5.2 Detection of allelic imbalance

The pipeline to detect allelic imbalance in both human and mouse data through germline heterozygous SNPs builds on previously published methods [14,33]. This pipeline consists of 4 steps: (1) count reads for a set of reference SNPs (human: 1000 Genomes Project [35] mouse: Mouse Genomes Project) and determine which SNPs are heterozygous based on the matched control sample. (2) Reconstruct haplotypes to improve the accuracy of the b-allele frequencies as is used in the Battenberg algorithm [33], this step is skipped for mouse samples due to the requirement of reference haplotype data. (3) Segmentation of the data in regions of constant signal. (4) Calling of segments with significantly different signal and multiple-testing correction.

Allele counts are obtained for each potential SNP. For a read to be included in the count both the mapping quality and the base quality at the position within the read have to meet the below thresholds shown in Supplementary Table 36. A single read is counted for fragments where read pairs overlap, selecting the highest quality read to be included. These thresholds are applied to all counts in both the samples of interest and the matched controls.

A SNP was considered to be heterozygous when it met the criteria listed in Supplementary Table 37 in the matched germline sample. These thresholds aim to select bona fide heterozygous SNPs. Lower coverage thresholds were applied to the human samples due to their lower effective sequencing coverage. To compensate for the lower certainty in these samples, we subsequently increased the penalty of starting a new segment (segmentation Gamma).

For human samples we next perform haplotype reconstruction to refine the BAF values, as is implemented in the Battenberg algorithm. Segmentation is subsequently performed via piecewise constant fitting, as implemented in the ASCAT software package [33], using the parameters listed in Supplementary Table 38. This step results in segments that each have the following values: genomic start and end position, mean BAF in the sample of interest and mean BAF in the matched control.

Finally, for each segment the BAF of the sample of interested is tested against the BAF of the matched control sample. The BAF is required to deviate 0.1 from 0.5 (i.e. BAF < 0.4 or BAF > 0.6) before a segment is tested, with the aim to prohibit noise from generating low quality calls. Testing is subsequently performed via a two-tailed t-test where the BAF of all heterozygous SNPs in the sample of interest are tested against the BAF of the same SNPs in the matched control. The resulting p-values are adjusted for multiple-testing (Bonferroni). A segment is considered having a BAF imbalance when the adjusted p-value from this test is below 0.05 (Supplementary Table 39).

6. Single cell RNA sequencing

6.1 Sample processing

YFPCreNotch1$^{flox/flox}$ mice were injected intraperitoneally (ip) with BNF at 80 mg.kg^{-1} and TAM at 1 mg and aged for 11 weeks. Uninduced littermates were used as wild type controls. Mouse esophagus was collected and placed immediately in PBS on ice. Esophagus was opened and cut into 4 equal pieces and placed in 1ml Dispase (2.4U/ml) at 37C for 10mins. Epithelia was then dissected away from the underlying stroma and muscle. Resulting epithelia was minced very finely using a scalpel and the slurry placed in 1ml trypsin-EDTA for 30mins at 37C. After incubation each sample was placed in 9ml ice-cold 0.04% BSA/PBS in a C-type GentleMacs tube (Miltenyi Biotec). Tissue was further dissociated using a GentleMACs dissociator with programs A1 and two rounds of program B1 with the sample kept on ice between programs. Samples were briefly centrifuged and filtered through a 30um filter (Miltenyi Biotec). Samples were then centrifuged at 4°C for 10mins at 500xg. The pellet was resuspended in ice-cold 0.04% BSA/PBS and centrifuged again a 4°C for 5mins at 300xg. Pellet was resuspended in ice-cold 0.04% BSA/PBS. Cells were counted and sufficient volume was placed into a Chromium reaction to allow 5000 cell recovery. Single cells were collected using Chromium (10X) with version 3 chemistry following manufacturer's instructions with one mouse esophagus per inlet. cDNA was sequenced using HiSeq4000 (Illumina) with the following read length 28 bp read 1; 91 bp read 2; 8 bp index 1; 0 bp index 2 with a single inlet per lane.

6.2 Alignment and transcript quantification

Alignment of the sequencing reads and expression quantification was performed for each library individually using the CellRanger pipeline version 3.0.2 (10xGenomics). We subsequently used EmptyDrops version 1.2.2 [36] to detect empty droplets in the raw feature count matrix output from

CellRanger and discarded any barcode identified as an empty droplet. All the subsequent analysis described below was performed in R version 4.1.3 (https://www.R-project.org/) using the Seurat software package version 4.0.3 [37].

6.3 Cell type detection

The pipeline to assign cell types first applies a number of filters per library to select healthy cells and remove any potential doublets. We filter on mitochondrial (MT) and overall expression levels: Cells are kept if their proportion of expression from MT genes is between 0.03 and 0.10, total expressed genes is between 2,500 and 6,500 and total expression is below 55,000 total unique molecule identifiers (UMI). Genes are kept if they are expressed in 30 cells or more per library.

After filtering, expression is normalized and adjusted for proportion of MT expression, total expression, total number of expressed genes and predicted cell cycle state using the SCTransform function from Seurat. So far, all steps have been applied to the four libraries individually. The libraries are next combined using Seurat's SCT integration methodology that aims to incorporate libraries, taking into account possible batch effects. To capture the majority of the variation we required 3,000 genes to be used for the integration. Next, principal component analysis (PCA) was performed and we selected the top 30 principal components to continue into Uniform Manifold Approximation and Projection (UMAP) dimensionality reduction, clustering of cells and detection of variable genes between clusters. A plot of the UMAP space revealed good mixing of the four libraries throughout (Fig. 4b).

Cell types were identified through a series of marker genes. For keratinocytes we used *Krt14*, *Tgm3*, and *Lor* and required a per cell cluster median of at least 50, 5 and 50 transcripts per million (TPM) respectively. For fibroblasts we used *Col1a1* with an average cluster TPM cut-off of greater than 10, for endothelial cells we used *Pecam1* requiring a median cluster TPM over 2 and, finally, for immune cells we used *Cd83*, *Cd84*, *Cd86*, *Cd52* and *Ptprc* with median cluster TPM values of 1, 1, 0.4, 50 and 0.25 respectively. Extended data Fig. 7b-d shows the expression of selected markers overlaid on the UMAP space. Extended data Fig. 7h-k shows expression of several keratinocyte marker genes.

Visual inspection of the assignment through a UMAP plot revealed that, even though the cell types were well separated (UMAP in Fig. 4c, Supplementary table 16), 35 cells had been marked as keratinocytes, but visually clustered with the other cell types. These cells were flagged and their cell type label was set to NA to reflect their ambiguous status. These cells were not used for further analysis.

The number of cells per cell type, per library were counted and divided by the total number of cells per library to produce the data shown in the bar graph in Fig. 4c.

6.4 Analysis of keratinocytes

Having identified which cells are keratinocytes, we restarted the analysis from the raw UMI counts, but now only including those cells that were previously marked as keratinocytes. The analysis differed from that above in two critical ways: 1. No cell cycle adjustment was performed as the cell cycle state and keratinocyte differentiation are deeply linked, with basal cells cycling and differentiating cells having exited the cell cycle, and 2. as now only keratinocytes were included, we

increased the minimum number of genes expressed per cell to 3,000 to strictly select for highly viable cells.

After integration of the four libraries, we performed a PCA and selected the top 10 principal components for further analysis. Clustering was performed and significant differentially expressed genes were identified through standard Seurat functions.

To determine the number of basal cells per library we made use of the visual dividing line in the UMAP space marked by a clear drop in expression of *Krt14* (basal cell marker gene) and the emergence of that of differentiation markers *Krt4* and *Tgm3* (Extended data Fig. 7h-k). The line separating the `circle` shape from the `tail` shape was defined to roughly capture the area where this expression divide manifests itself. We subsequently counted the cells from each library on both sides of the line to obtain the data shown in Fig. 4e (Supplementary table 25). We note that the exact placement of the dividing line matters little for the differences in proportions between libraries, because of the good mixing of cells from both wild-type and knock out libraries across the UMAP space (Fig. 4d).

The Seurat analysis pipeline performs clustering via the Leiden algorithm to partition cells in similar groups (Fig. 4g). We show that these clusters correspond to previously reported findings, using the same marker genes [38]. Fig. 4f shows the Seurat processed expression values for these markers of all cells.

6.5 Cell condition density analysis using Milo

We applied the Milo algorithm to formally evaluate whether the *Notch1* knockout keratinocytes present a significant difference in cell abundance (Fig. 4d)[39]. The main advantage of using Milo over testing the number of cells per cluster and condition is that it does not rely on clustering information. The established UMAP space for keratinocytes shows cells form a deeply integrated continuum for both cycling and differentiating states as well as the transition between the two. Clusters form arbitrary boundaries in such a scenario, hence the application of an algorithm that looks for a difference in cell density between the experimental conditions.

We loaded the keratinocyte data, processed via Seurat as described above, transformed it into a SingCellExperiment object and subsequently created a Milo object directly from it using the Milo constructor. The experimental condition for each library was set to either KO (knockout) or WT (wild type) to set Milo up for testing between them. The Milo pipeline was then applied with parameters according to the Milo demo (https://marionilab.github.io/miloR/articles/milo_demo.html).

The demo suggests to set the k parameter (the number of cells considered as neighbors) such that the neighborhood size distribution has a peak between 50 and 100 to maximize testing statistical power. We set k=30 to obtain a peak between 50 and 75 and the overwhelming volume of the distribution between 50 and 100. The test showed no significant difference (absence of colored circles).

Supplementary note references

1. Doupe, D.P. *et al.* A single progenitor population switches behavior to maintain and repair esophageal epithelium. *Science* **337**, 1091-3 (2012).
2. Clayton, E. *et al.* A single type of progenitor cell maintains normal epidermis. *Nature* **446**, 185-189 (2007).
3. Klein, A.M., Brash, D.E., Jones, P.H. & Simons, B.D. Stochastic fate of p53-mutant epidermal progenitor cells is tilted toward proliferation by UV B during preneoplasia. *Proceedings of the National Academy of Sciences* **107**, 270-275 (2010).
4. Murai, K. *et al.* Epidermal Tissue Adapts to Restrain Progenitors Carrying Clonal p53 Mutations. *Cell Stem Cell* **23**, 687-699.e8 (2018).
5. Hall, M.W.J., Jones, P.H. & Hall, B.A. Relating evolutionary selection and mutant clonal dynamics in normal epithelia. *Journal of the Royal Society Interface* **16**, 20190230 (2019).
6. Colom, B. *et al.* Spatial competition shapes the dynamic mutational landscape of normal esophageal epithelium. *Nature Genetics* **52**, 604–614 (2020).
7. Wright, S. Evolution in Mendelian Populations. *Genetics* **16**, 97-159 (1931).
8. Fowler, J.C. *et al.* Selection of oncogenic mutant clones in normal human skin varies with body site. *Cancer Discovery*, CD-20-1092 (2020).
9. Tavaré, S., Balding, D.J., Griffiths, R.C. & Donnelly, P. Inferring coalescence times from DNA sequence data. *Genetics* **145**, 505-18 (1997).
10. Beaumont, M.A., Zhang, W. & Balding, D.J. Approximate Bayesian computation in population genetics. *Genetics* **162**, 2025-35 (2002).
11. Smirnov, N.V. Estimate of deviation between empirical distribution functions in two independent samples. *Bull Moscow University* **2**, 3-16 (1939).
12. Toni, T., Welch, D., Strelkowa, N., Ipsen, A. & Stumpf, M.P. Approximate Bayesian computation scheme for parameter inference and model selection in dynamical systems. *J R Soc Interface* **6**, 187-202 (2009).
13. Klinger, E., Rickert, D. & Hasenauer, J. pyABC: distributed, likelihood-free inference. *Bioinformatics* **34**, 3591-3593 (2018).
14. Martincorena, I. *et al.* Somatic mutant clones colonize the human esophagus with age. *Science* **362**, 911-917 (2018).
15. Radtke, F. *et al.* Deficient T cell fate specification in mice with an induced inactivation of Notch1. *Immunity* **10**, 547-58 (1999).
16. Rand, M.D. *et al.* Calcium depletion dissociates and activates heterodimeric notch receptors. *Mol Cell Biol* **20**, 1825-35 (2000).
17. Anders, S., Pyl, P.T. & Huber, W. HTSeq--a Python framework to work with high-throughput sequencing data. *Bioinformatics* **31**, 166-9 (2015).
18. Dobin, A. *et al.* STAR: ultrafast universal RNA-seq aligner. *Bioinformatics* **29**, 15-21 (2013).
19. Love, M.I., Huber, W. & Anders, S. Moderated estimation of fold change and dispersion for RNA-seq data with DESeq2. *Genome Biol* **15**, 550 (2014).
20. Yu, G., Wang, L.G., Han, Y. & He, Q.Y. clusterProfiler: an R package for comparing biological themes among gene clusters. *Omics* **16**, 284-7 (2012).
21. Huang da, W., Sherman, B.T. & Lempicki, R.A. Systematic and integrative analysis of large gene lists using DAVID bioinformatics resources. *Nat Protoc* **4**, 44-57 (2009).
22. Subramanian, A. *et al.* Gene set enrichment analysis: a knowledge-based approach for interpreting genome-wide expression profiles. *Proc Natl Acad Sci U S A* **102**, 15545-50 (2005).
23. Anders, S. & Huber, W. Differential expression analysis for sequence count data. *Genome Biology* **11**, R106 (2010).

24. Jones, D. *et al.* cgpCaVEManWrapper: Simple Execution of CaVEMan in Order to Detect Somatic Single Nucleotide Variants in NGS Data. *Current Protocols in Bioinformatics* **56**, 15.10.1-15.10.18 (2016).
25. Raine, K.M. *et al.* cgpPindel: Identifying Somatically Acquired Insertion and Deletion Events from Paired End Sequencing. in *Current protocols in bioinformatics* Vol. 52 15.7.1-15.7.12 (2015).
26. Gerstung, M., Papaemmanuil, E. & Campbell, P.J. Subclonal variant calling with multiple samples and prior knowledge. *Bioinformatics (Oxford, England)* **30**, 1198-1204 (2014).
27. Martincorena, I. *et al.* Tumor evolution. High burden and pervasive positive selection of somatic mutations in normal human skin. *Science* **348**, 880-6 (2015).
28. Li, H. Aligning sequence reads, clone sequences and assembly contigs with BWA-MEM. *ArXiv*, 1303.3997 (2013).
29. Menzies, A. *et al.* VAGrENT: Variation Annotation Generator. *Current Protocols in Bioinformatics* **52**, 15.8.1-15.8.11 (2015).
30. Martincorena, I. *et al.* Universal Patterns of Selection in Cancer and Somatic Tissues. *Cell* **171**, 1029-1041.e21 (2017).
31. Keane, T.M. *et al.* Mouse genomic variation and its effect on phenotypes and gene regulation. *Nature* **477**, 289-94 (2011).
32. Scheinin, I. *et al.* DNA copy number analysis of fresh and formalin-fixed specimens by shallow whole-genome sequencing with identification and exclusion of problematic regions in the genome assembly. *Genome Res* **24**, 2022-32 (2014).
33. Nik-Zainal, S. *et al.* The life history of 21 breast cancers. *Cell* **149**, 994-1007 (2012).
34. Kuilman, T. *et al.* CopywriteR: DNA copy number detection from off-target sequence data. *Genome Biol* **16**, 49 (2015).
35. McVean, G.A. *et al.* An integrated map of genetic variation from 1,092 human genomes. *Nature* **491**, 56-65 (2012).
36. Lun, A.T.L. *et al.* EmptyDrops: distinguishing cells from empty droplets in droplet-based single-cell RNA sequencing data. *Genome Biology* **20**, 63 (2019).
37. Stuart, T. *et al.* Comprehensive Integration of Single-Cell Data. *Cell* **177**, 1888-1902.e21 (2019).
38. McGinn, J. *et al.* A biomechanical switch regulates the transition towards homeostasis in oesophageal epithelium. *Nat Cell Biol* **23**, 511-525 (2021).
39. Dann, E., Henderson, N.C., Teichmann, S.A., Morgan, M.D. & Marioni, J.C. Differential abundance testing on single-cell data using k-nearest neighbor graphs. *Nat Biotechnol* **40**, 245-253 (2022).
