# Supplementary material for: Notch1 mutations drive clonal expansion in normal esophageal epithelium but impair tumor growth
Source: Nat Genet. 2023 Jan 19;55(2):232–45. doi: 10.1038/s41588-022-01280-z (PMC9925379; doi:10.1038/s41588-022-01280-z)
Supplement: Supplementary file 2 — Reporting Summary [file 41588_2022_1280_MOESM2_ESM.pdf]

nature research

Corresponding author(s): Philip H Jones

Last updated by author(s): Nov 27, 2022

Reporting Summary

Nature Research wishes to improve the reproducibility of the work that we publish. This form provides structure for consistency and transparency in reporting. For further information on Nature Research policies, see our Editorial Policies and the Editorial Policy Checklist.

Statistics

For all statistical analyses, confirm that the following items are present in the figure legend, table legend, main text, or Methods section.

n/a	Confirmed	
☐	☒	The exact sample size (*n*) for each experimental group/condition, given as a discrete number and unit of measurement
☐	☒	A statement on whether measurements were taken from distinct samples or whether the same sample was measured repeatedly
☐	☒	The statistical test(s) used AND whether they are one- or two-sided<br>*Only common tests should be described solely by name; describe more complex techniques in the Methods section.*
☐	☒	A description of all covariates tested
☐	☒	A description of any assumptions or corrections, such as tests of normality and adjustment for multiple comparisons
☐	☒	A full description of the statistical parameters including central tendency (e.g. means) or other basic estimates (e.g. regression coefficient) AND variation (e.g. standard deviation) or associated estimates of uncertainty (e.g. confidence intervals)
☐	☒	For null hypothesis testing, the test statistic (e.g. *F*, *t*, *r*) with confidence intervals, effect sizes, degrees of freedom and *P* value noted<br>*Give P values as exact values whenever suitable.*
☒	☐	For Bayesian analysis, information on the choice of priors and Markov chain Monte Carlo settings
☒	☐	For hierarchical and complex designs, identification of the appropriate level for tests and full reporting of outcomes
☒	☐	Estimates of effect sizes (e.g. Cohen's *d*, Pearson's *r*), indicating how they were calculated

Our web collection on statistics for biologists contains articles on many of the points above.

Software and code

Policy information about availability of computer code

Data collection	Confocal images were obtained using acquisition software Leica Application Suite X (LAS X). Confocal Z stack images were rendered and analyzed with Volocity 6 Software (Perkin Elmer). Hematoxylin and eosin (H&E) stained tissues were rendered and analyzed with NanoZoomer Digital Pathology software (NDP.view2, Hamamatsu). Immune capillary electrophoresis was performed and analyzed using Compass for SW version 4.1.0. qPCR data were obtained using StepOne Software v2.3.
Data analysis	Confocal images were analyzed using Volocity 6 Software (Perkin Elmer). Hematoxylin and eosin (H&E) stained tissues were analyzed with NanoZoomer Digital Pathology software (NDP.view2, Hamamatsu). Immune capillary electrophoresis was analyzed using Compass for SW version 4.1.0. qPCR data were analyzed using StepOne Software v2.3. GraphPad Prism 8.3.1. was used for data plotting and statistical analysis. Python package Scipy 1.7.3 (https://scipy.org/citing-scipy/) was also used for statistics. For DNA sequencing, paired-end reads were aligned with BWA-MEM (v.0.7.17, https://github.com/lh3/bwa) with optical and PCR duplicates marked using Biobambam2 (v.2.0.86, https://gitlab.com/german.tischler/biobambam2). For clonal datasets, substitution mutations were called using the CaVEMan (Cancer Variants through Expectation Maximization, version 1.13.14) variant caller (http://cancerit.github.io/CaVEMan). Insertions and deletions were called using cgpPindel (http://cancerit.github.io/cgpPindel, version 3.3.0). Mutations were annotated using VAGrENT (https://github.com/cancerit/VAGrENT, version 3.3.3). For subclonal calling in highly mutagenized tissue, we used ShearwaterML algorithm from the deepSNV package (v1.21.3, https://github.com/gerstung-lab/deepSNV). We used the maximum-likelihood implementation of the dNdScv algorithm (v0.0.1.0, https://github.com/im3sanger/dndscv) to identify genes under positive selection.<br>For RNAseq, the alignment files were sorted and duplicate-marked using Biobambam2 2.0.54, and the read summarization performed by the htseq-count script from version 0.6.1p1 of the HTSeq framework (Anders et al., 2015; Dobin et al., 2013). Differential gene expression was analyzed using the DESeq2 R package version 1.20.0 (Love et al., 2014), and the downstream pathway analysis and visualization using R version 3.5.3 (https://www.R-project.org/) and the packages Pheatmap version 1.0.12 (https://cran.r-project.org/package=pheatmap), RColorBrewer version 1.1.2 (https://cran.r-project.org/package=RColorBrewer), clusterProfiler version 3.8.1 (Yu et al., 2012) and org.Mm.eg.db version 3.6.0 (https://bioconductor.org/packages/org.Mm.eg.db/). For Gene Set Enrichment Analysis (GSEA), we used GO BP or KEGG gene sets v7.5.1 from the Molecular Signature Database (MSigDB) in GSEA software v4.2.3. (Subramanian et al. 2005). The functions of the DEGs from DESeq2 analysis were annotated using the database for annotation, visualization, and integrated discovery (DAVID v6.8)

(Huang da et al., 2009).
For sc-RNAseq, alignment of the sequencing reads and expression quantification was performed for each library individually using the CellRanger pipeline version 3.0.2 (10xGenomics). We subsequently used EmptyDrops version 1.2.2 to detect empty droplets in the raw feature count matrix output from CellRanger and discarded any barcode identified as an empty droplet. All the subsequent analysis described was performed in R version 4.1.3 (https://www.R-project.org/) using the Seurat software package version 4.0.3.
Custom codes for clone simulations, for copy number analysis and sc-RNAseq analyses are available at https://github.com/PHJonesGroup/Abby_etal_SI_code

For manuscripts utilizing custom algorithms or software that are central to the research but not yet described in published literature, software must be made available to editors and reviewers. We strongly encourage code deposition in a community repository (e.g. GitHub). See the Nature Research guidelines for submitting code & software for further information.

Data

Policy information about availability of data

All manuscripts must include a data availability statement. This statement should provide the following information, where applicable:

- Accession codes, unique identifiers, or web links for publicly available datasets
- A list of figures that have associated raw data
- A description of any restrictions on data availability

Accession numbers for the datasets are as follows:-Targeted sequencing of Human esophageal epithelium microbiopsies (EGA): EGAD00001006969. -Targeted sequencing of aged Notch1 +/- mouse esophageal epithelium microbiopsies (ENA): ERP126992-Targeted sequencing of mouse normal esophageal epithelium 28 weeks after DEN SOR treatment (ENA): ERP126993 -Targeted sequencing of mouse esophageal tumours 28 weeks after DEN SOR treatment (ENA): ERP126994-Transcriptomic analysis of Notch1 mutant esophageal epithelium (ENA): ERP126995 -Single cell transcriptional analysis of Notch1 mutant esophageal epithelium (ENA): ERP126996.- Transcriptomic analysis of Notch1 mutant esophageal tumors and adjacent normal tissue 28 weeks after DEN SOR treatment (ENA): ERP137375.
All data displayed in the figures are available in Supplementary tables 2-30.
The codes developed in this study has been made publicly available and can be found at https://github.com/PHJonesGroup/Abby_etal_SI_code

Field-specific reporting

Please select the one below that is the best fit for your research. If you are not sure, read the appropriate sections before making your selection.

☒ Life sciences ☐ Behavioural & social sciences ☐ Ecological, evolutionary & environmental sciences

For a reference copy of the document with all sections, see nature.com/documents/nr-reporting-summary-flat.pdf

Life sciences study design

All studies must disclose on these points even when the disclosure is negative.

Sample size

Sample size was not predetermined by statistical methods. Sample size was determined by pilot studies for lineage tracing and clonal sequencing studies and by previously published studies for highly mutagenized sequencing and carcinogenesis studies.

Data exclusions

Data exclusion was only made in RNA sequencing studies to improve the quality of the datasets.
Quality control of RNA-seq study: 'Transcriptomic analysis of Notch1 mutant esophageal tumors and adjacent normal tissue 28 weeks after DEN SOR treatment ' revealed one outlier sample on PCA plot out of the initial 36 samples dataset. The outlier sample was removed from the analysis. Quality control of RNA-seq study: 'Transcriptomic analysis of Notch1 mutant esophageal epithelium' revealed an outlier control sample on PCA plot but deep analysis of the noise identified 151 genes with aberrant signal within the control group. Thorough checks with complete analysis were performed with and without these genes and/ or the sample, leading to the conclusion that none of these actions modified the conclusions of the analysis but excluding these genes and not the affected control sample for final analysis preserved the most data and resulted in removing 7 false positive hits. For 'Single cell transcriptional analysis of Notch1 mutant esophageal epithelium', poor quality cells were filtered out as is considered best practice. Details are provided in respective Methods and Supplementary Note.

Replication

For Human histological study, three donors were analyzed per age group (young, middle-aged and elderly). For Human sequencing, we analyzed multiple biopsies from 6 distinct donors. For mouse studies, experiments were performed with at least 3 mice per time point constituting 3 independent biological replicates except on two occasions. scRNA-seq was performed on two biological replicates per genotype as each tissue yielded sequencing data from thousands of cells. Findings were further verified in separate experiments (Immunostaining and EdU/BrdU lineage tracing in young mice; Immunostaining and EdU in aged mice) involving 3 to 4 biological replicates. Pilot neutralizing antibody titration involved some dosing that were not repeated but the final dosage was confirmed with 3 biological replicates. All attempts at replicating the findings from the study were successful.

Randomization

For mouse experiments, mice of relevant genotype were randomly assigned to each experimental protocol. For Human study, donors were randomly assigned for sequencing/ histological analysis based on their age at tissue collection.

Blinding

Blinding was performed but its feasibility was sometimes limited. Technicians and investigators were blinded to group allocation during mice treatments, except when performing treatments that required such information (high inductions, antibody treatments). Samples were systematically given identification numbers so that investigators were blinded to genotype or treatment during processing and analysis when this was applicable. Blinding was not applicable or effective when the information of material were required for analysis or when the experiments required sampling using immunostaining that reflected the genotype.

Reporting for specific materials, systems and methods

We require information from authors about some types of materials, experimental systems and methods used in many studies. Here, indicate whether each material, system or method listed is relevant to your study. If you are not sure if a list item applies to your research, read the appropriate section before selecting a response.

Materials & experimental systems

n/a	Involved in the study
☐	☒ Antibodies
☒	☐ Eukaryotic cell lines
☒	☐ Palaeontology and archaeology
☐	☒ Animals and other organisms
☐	☒ Human research participants
☒	☐ Clinical data
☒	☐ Dual use research of concern

Methods

n/a	Involved in the study
☒	☐ ChIP-seq
☒	☐ Flow cytometry
☒	☐ MRI-based neuroimaging

Antibodies

Antibodies used

Unconjugated primary antibodies
Protein (clone) species and clonality Company Reference Dilution
KRT14 chicken polyclonal Biolegend 906001 1/1000
KRT14 rabbit polyclonal Covance PRB-155P 1/1000
Ki67 (SP6) rabbit monoclonal Abcam ab16667 1/500
KRT6a (Poly19057) rabbit polyclonal Biolegend PRB-169P 1/1000
Ki67 (MIB1) mouse monoclonal Agilent M724029-2 1/500
NOTCH1 (D1E11) rabbit monoclonal Cell Signaling Technology 3608 1/200-1/1000
GFP chicken polyclonal Invitrogen A10262 1/500
Loricrin (AF 62) rabbit polyclonal Covance PRB-145P 1/2000
Keratin 4 (6B10) mouse monoclonal Vector Labs VP-C399 1/1000
NICD1 (Val1744; D3B8) rabbit monoclonal Cell Signaling Technology4147 1/100
E-Cadherin (24E10) rabbit monoclonal Cell Signaling Technology 3195 1/500
CD45 (30-F11) rat monoclonal Biolegend 103102 1/200
CD31 ((MEC7.4) rat monoclonal Abcam ab7388 1/200
cleaved caspase 3 rabbit polyclonal Abcam ab2302 1/200
BrdU [BU1/75 (ICR1)] rat monoclonal Abcam ab6326 1/250
CCNB1 rabbit polyclonal Cell Signaling Technology4138 1/200
ZEB1 (E2G6Y) rabbit monoclonal Cell Signaling Technology70512 1/500
Phospho-Erk1/2 -Thr202/Tyr204 (D13.14.4E) rabbit monoclonal Cell Signaling Technology 4370 1/200
Erk1/2 (137F5) rabbit monoclonal Cell Signaling Technology 4695 1/200

Conjugated primary antibodies
Protein/fluorophore Conjugation method Company Reference Dilution
ITGA6-Alexa 647 Company Biolegend 313610 1/200
Phalloidine-Alexa 488 Company Invitrogen A12379 1/200
WGA-Alexa 647 Company Invitrogen W32466 1/500
K6a-Alexa 555 Thermo Fisher labeling kit (A20187) Biolegend PRB-169P 1/500
Histone H3 -phospho S10- Alexa 647 Alexa Fluor® 647 (Company) Abcam ab196698 1/10000

Seconday antibodies
Host/Fluorophore Target Company Reference Dilution
Donkey Alexa-555 anti-Rabbit Invitrogen A31572 1/500
Donkey Alexa-488 anti-rabbit Invitrogen A21206 1/500
Donkey Alexa-647 anti-rabbit Invitrogen A31573 1/500
Donkey Alexa-488 anti-chicken Jackson 703-545-155 1/250
Donkey Alexa-488 anti-mouse Invitrogen A21202 1/500
Goat Alexa-555 anti-rat Invitrogen A21434 1/500
Donkey Alexa-647 anti-mouse Invitrogen A31571 1/500
Donkey Alexa-488 anti-rat Invitrogen A21208 1/500

Validation

NOTCH1 (D1E11) rabbit monoclonal was validated for immunofluorescence, immunohistochemistry and Immune Capillary Electrophoresis in our study using a knock-out NOTCH1 mouse model. Furthermore, the protein conservation between Human and Mouse is very high and immunofluorescence and immunohistochemistry assays in Human tissues revealed areas showing both expression and absence of expression within the same tissues, further confirmed being mutant areas by DNA sequencing. NICD1 (Val1744; D3B8) rabbit monoclonal antibody is a highly cited antibody (https://www.cellsignal.co.uk/products/primary-antibodies/cleaved-notch1-val1744-d3b8-rabbit-mab/4147) and we also validated it both in immunofluorescence and Immune Capillary Electrophoresis in our study using a knock-out mouse model. BrdU [BU1/75 (ICR1)] is a highly cited rat monoclonal antibody, suitable for immunofluorescence (https://www.abcam.com/brdu-antibody-bu175-icr1-proliferation-marker-ab6326.html). Alexa

Fluor® 647 Anti-Histone H3 (phospho S10) antibody [mAbcam 14955] (ab196698) is the conjugated version of highly cited mouse monoclonal ab14955 (https://www.abcam.com/histone-h3-phospho-s10-antibody-mabcam-14955-ab14955.html). ZEB1 (E2G6Y) #70512 rabbit monoclonal antibody is validated in immunofluorescence by Cell Signaling (https://www.cellsignal.com/products/primary-antibodies/zeb1-e2g6y-xp-rabbit-mab/70512). Cyclin B1 Antibody #4138 is a highly cited and validated antibody from Cell Signaling (https://www.cellsignal.com/products/primary-antibodies/cyclin-b1-antibody/4138). Phospho-Erk1/2 #4370 is a highly cited and validated antibody from Cell signaling (https://www.cellsignal.com/products/primary-antibodies/phospho-p44-42-mapk-erk1-2-thr202-tyr204-d13-14-4e-xp-rabbit-mab/4370). Total Erk1/2 #4695 is a highly cited and validated antibody from Cell Signaling (https://www.cellsignal.com/products/primary-antibodies/p44-42-mapk-erk1-2-137f5-rabbit-mab/4695). All other primary antibodies were used for immunofluorescence, validation is described in PMID: 17330052, PMID: 22821983, PMID: 24814514 and PMID: 27548914.

Animals and other organisms

Policy information about studies involving animals; ARRIVE guidelines recommended for reporting animal research

Laboratory animals	Laboratory animals were mice from C57BL/6J background or from a transgenic mixed C57BL/6J and 129X1/SvJ background (YFPCreNotch1). Strains used were as indicated for each experiments: Rosa26floxedYFPAhCreERTNotch1flox (YFPCreNotch1 with Notch1 genotype status precised for each experiment) and C57Bl/6.<br>Mouse housing was carried out in individually ventilated cages (19-23°C, RH55%±10%, 12/12 light dark cycle, 15-20 air changes per hour). Mice were fed on standard chow. Mice were maintained on a specific and opportunistic pathogen free health status and were immune competent. Animals were not involved in any previous experiments. Both male and female adult mice at 10-16 weeks of age at the start of the experiments were used.
Wild animals	The study did not involve wild animals
Field-collected samples	The study did not involve samples collected from the field
Ethics oversight	UK government Home Office project licences, which include stringent local and government ethical review. UK Home Office Project Licenses 70/7543, P14FED054 or PF4639B40.

Note that full information on the approval of the study protocol must also be provided in the manuscript.

Human research participants

Policy information about studies involving human research participants

Population characteristics	4 female and 6 male Human donors, aged 20 to 78 years old.
Recruitment	Esophageal tissue was obtained from deceased organ donors from whom organs were being retrieved for transplantation. Informed consent was obtained from next of kin. Consecutive cases were recruited. There was no self selection bias. The sample is likely to be representative of organ transplant donor population in Eastern England.
Ethics oversight	Informed consent for the use of tissue was obtained from the donor's relatives (REC reference: 15/EE/0152 NRES Committee East of England - Cambridge South).

Note that full information on the approval of the study protocol must also be provided in the manuscript.
