# Supplementary material for: Notch1 mutations drive clonal expansion in normal esophageal epithelium but impair tumor growth
Source: Nat Genet. 2023 Jan 19;55(2):232–45. doi: 10.1038/s41588-022-01280-z (PMC9925379; doi:10.1038/s41588-022-01280-z)
Supplement: Source Data Extended Data Figs. 3h and 9b,c — Visual representations of immune capillary electrophoresis data from Extended Data Figs. 3h and 9b,c in the form of virtual gels. [file 41588_2022_1280_MOESM7_ESM.pdf]

Visual representations of Immune Capillary Electrophoresis from Extended data Fig. 3h

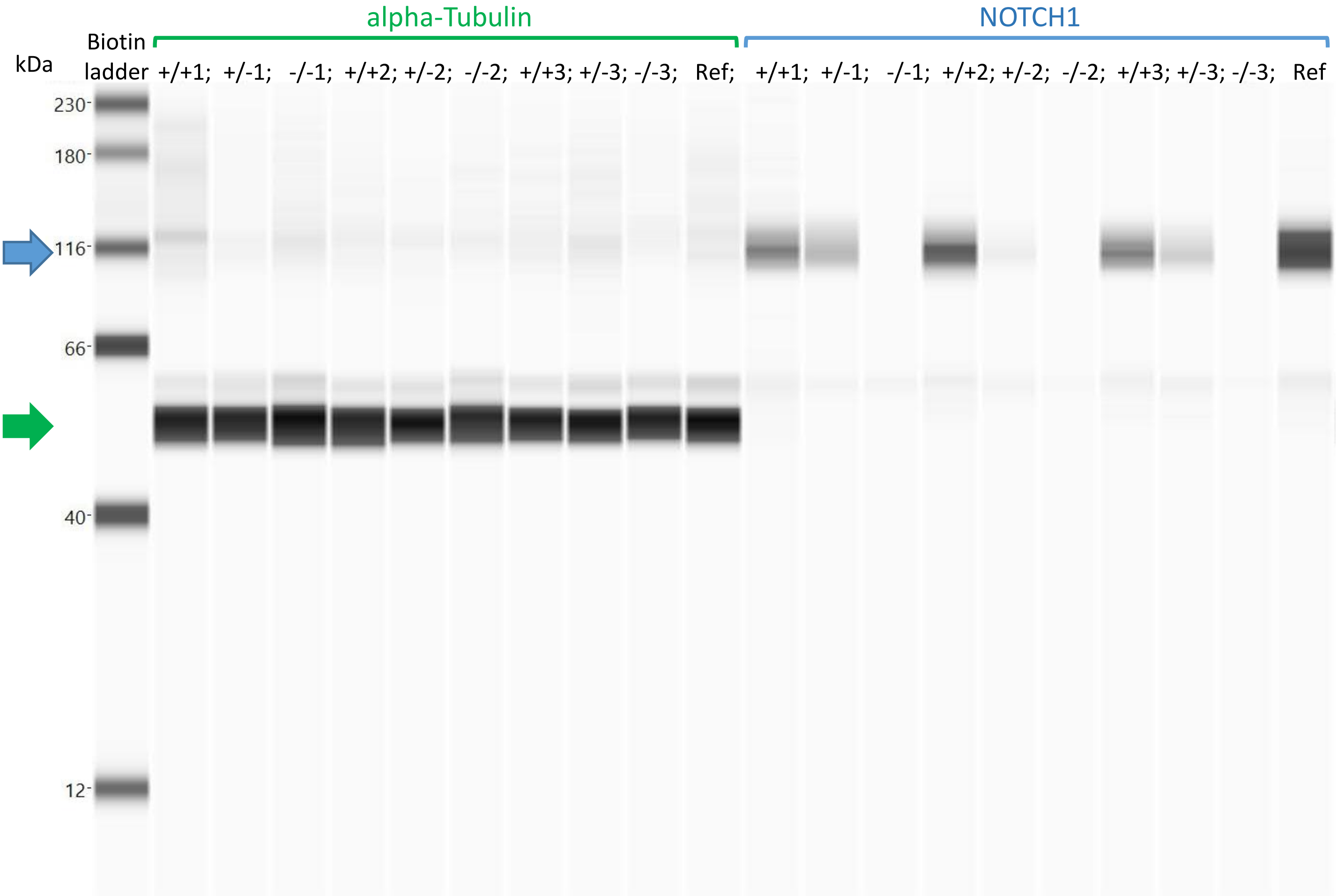

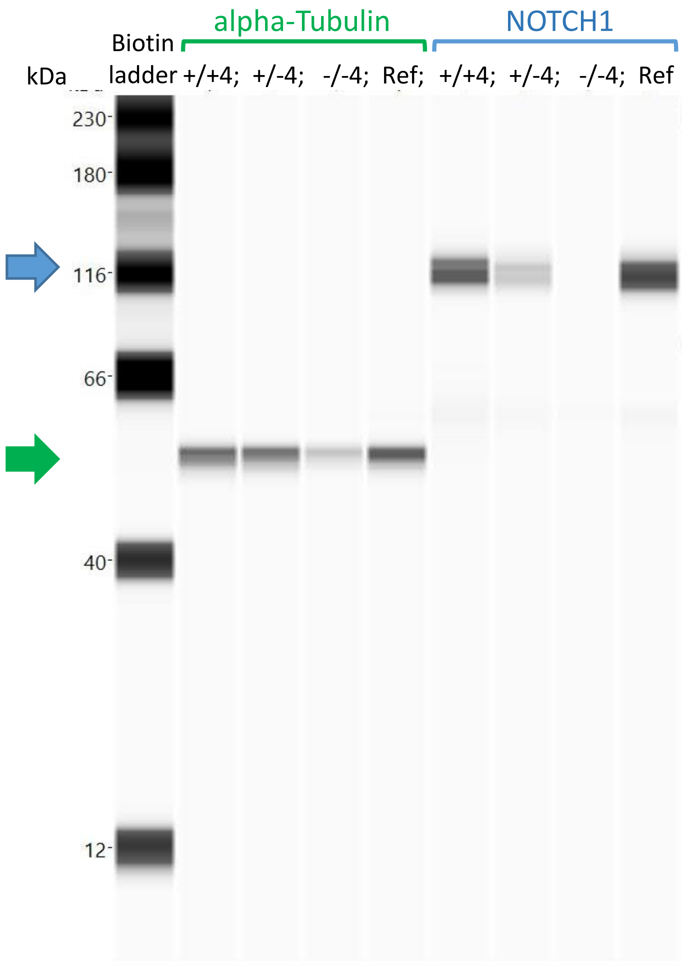

These are visual representations of Immune Capillary Electrophoresis performed using Wes Simple^{TM} (ProteinSimple) following manufacturer's instructions and analyzed using Compass for SW version 4.1.0. Areas of peaks at the appropriate protein size were quantified. For each sample NOTCH1 signal at 116kDa (NICD1+ NTM1) was normalized by alpha-Tubulin signal at 55kDa. Normalization was performed within the same assay. Wild type samples were analyzed within the same assays as the Notch1+/- and Notch1-/- samples. Replicates n1 to n3 were analyzed in the same assay, replicates n4 were analyzed in a separate assay and data combined using an external reference (Ref).

Data are shown in Supplementary Table 6.

Visual representations of Immune Capillary Electrophoresis from Extended data Fig. 9b,c

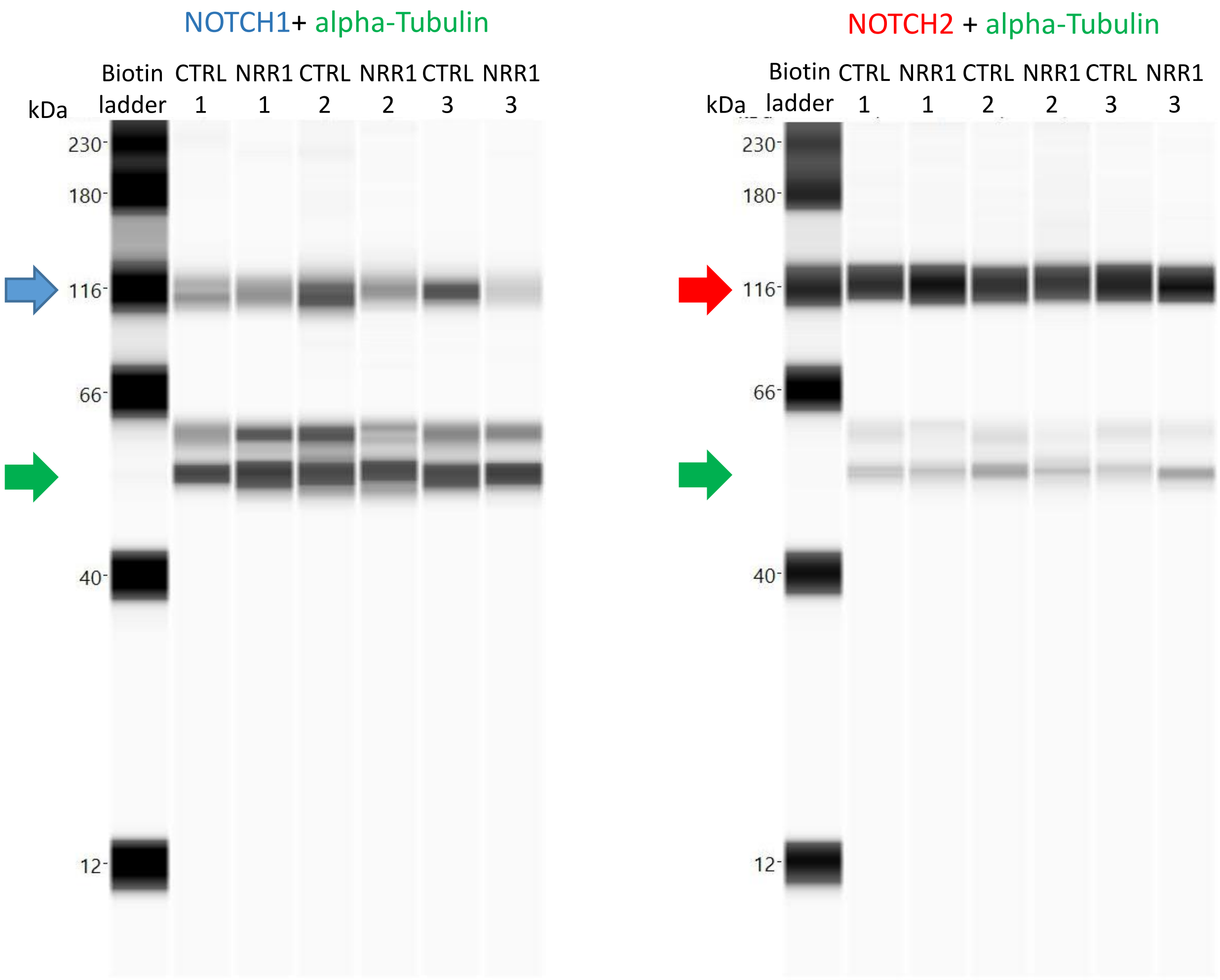

These are visual representations of Immune Capillary Electrophoresis performed using Wes Simple™ (ProteinSimple) following manufacturer's instructions and analyzed using Compass for SW version 4.1.0.

Areas of peaks at the appropriate protein size were quantified. For each sample NOTCH1 signal at 116kDa (NICD1+ NTM1), or NOTCH2 signal (NICD2+NTM2) at 117-119kDa, was normalized by alpha-Tubulin signal at 55kDa. Normalization was performed within the same assay. Data are shown in Supplementary Table 25.
